# Relationship between Estimated Glomerular Filtration Rate and Cardiovascular Mortality in a Japanese Cohort with Long-Term Follow-Up

**DOI:** 10.1371/journal.pone.0156792

**Published:** 2016-06-06

**Authors:** Kei Nagai, Toshimi Sairenchi, Fujiko Irie, Hiroshi Watanabe, Hitoshi Ota, Kunihiro Yamagata

**Affiliations:** 1 Department of Nephrology, Faculty of Medicine, University of Tsukuba, Tennodai, Tsukuba, Ibaraki, Japan; 2 Department of Public Health, Dokkyo Medical University School of Medicine, Shimotsugagun-Mibu, Japan; 3 Ibaraki Health Plaza, Ibaraki Health Service Association, Mito, Japan; 4 Department of Health and Welfare, Ibaraki Prefectural Office, Mito, Japan; 5 Ibaraki Health Service Association, Mito, Japan; Fondazione G. Monasterio, ITALY

## Abstract

**Background:**

Patients with renal impairment are at risk of not only end-stage kidney disease but also cardiovascular disease (CVD). The current definition of CKD stage G3a is eGFR 45–59 ml/min/1.73 m^2^ and of G3b is 30–44 ml/min/1.73 m^2^, and subjects in the CKD 3a category are considered to be at lower risk of mortality than are those in CKD 3b.

**Methods:**

We evaluated the outcome of 97,043 people (33,131 men and 63,912 women) living in Ibaraki Prefecture who underwent annual community-based health checkups beginning in 1993 at age 40–80 years and who were followed for a mean of 17.1 years.

**Results:**

The number of all-causes deaths was 20,534 (10,375 men and 10,159 women), of which 5,995 (2,695 men and 3,300 women) were deaths due to CVD. Multivariable-adjusted hazard ratio for CVD death in the eGFR 45–49 ml/min/1.73 m^2^ category was significantly increased (1.82; 95% confidential interval, 1.23–2.69) in non-elderly men, whereas all-cause mortality and CVD mortality in elderly men with eGFR 45–49 ml/min/1.73m^2^ were non significant. In contrast, both in non-elderly women and in elderly women with eGFR 45–49 ml/min/1.73 m^2^ showed small, but significant, increases in the risks of all-cause mortality and CVD.

**Conclusions:**

We demonstrated proportionate increases in mortality with decreasing eGFR in a Japanese CKD population. Like patients in the CKD G3b subgroup, non-elderly men and women with an eGFR of 45–49 ml/min/1.73 m^2^ (i.e. a part of CKD G3a) are at considerable risk of CVD mortality. Age dependent and eGFR dependent finer risk recognition were required for CVD prevention in clinical practice with regard to CKD patients.

## Introduction

The prevalence of chronic kidney disease (CKD) stage 3 to 5 (G3–G5) is estimated to be about 13% in the Japanese general population [1, 2]. In the 2009 CKD guidebook [3], the Japanese Societies of Nephrology (JSN) recommended the referral of all adult CKD patients (age 20 years or older) whose eGFR was <50 mL/min/1.73 m^2^ to nephrologists, because an epidemiological study [4] indicated that the major part of CKD patients have 50–59 ml/min/1.73m^2^ of eGFR. In a cohort of Department of Veterans Affairs patients aged 18 to 100 years of age, the association of eGFR with mortality was weaker in elderly than in younger patients: whereas severe reductions in eGFR (that is, to 40–49 ml/min/1.73 m^2^) were associated with an increased risk of death in all age groups, reductions in eGFR to 50–59 ml/min/1.73 m^2^ were associated with an increased adjusted risk of death only among patients who were younger than 65 years [5].

The KDIGO guidelines advocated the categorization of CKD according to renal and cardiovascular outcomes [6], in light of the results of international clinical epidemiology studies [7]. In this regard, CKD stage G3 was subdivided into G3a and G3b by applying a cutoff point of eGFR 45 ml/min/1.73 m^2^ for both CVD and ESKD risk assessment [7]. Using this category, subjects with CKD G3a with their eGFR 45–49 ml/min/1.73 m^2^ regarded as low risk population comparing to subjects with CKD G3b. Furthermore, in 2012, the JSN recommended that non-elderly people (age, 40–69 years) with eGFR >50 ml/min/1.73 m^2^ and elderly people (older than 70 years) with eGFR >40 ml/min/1.73 m^2^ need not be referred to nephrologists for follow-up unless their proteinuria exceeded 0.5 g daily [8]. To address this issue, we aimed to evaluate CVD events among Japanese men and women with CKD, especially with G3a and G3b, in a large cohort with long-time follow-up.

## Materials and Methods

### Study population

The initial study population comprised 97,043 people (33,131 men and 63,912 women) living in Ibaraki Prefecture who aged 40–80 years when they first participated in annual community-based health checkups, in 1993 [9]. People with a history of CVD (2,128 men and 3,114 women) and with missing data (638 men and 1,616 women) were excluded from the study (**S1 Table**); the data from the remaining 89,547 people (30,365 men [mean age, 60.2 years] and 59,182 women [57.8 years]) were analyzed. The population analyzed comprised 13,408 elderly people (70–80 years old) and 76,139 non-elderly people (40–69 years old), with a mean follow-up of 17.1 years (range, 0.1 to 19.7 years) and overall follow-up of 1,532,938 person–years. This study was conducted according to the guidelines of the Declaration of Helsinki, and the study protocol was approved by the Ethics Committee of Ibaraki Prefectural Office.

### Mortality surveillance

Mortality surveillance was conducted with systematic review of the death certificates and resident registration in cooperation with public health centers and municipal government offices. The details regarding the methods for mortality surveillance have been reported previously [9]. Briefly, the underlying causes of death were coded according to the International Classification of Diseases, 9th revision (ICD-9, 1993–1994) and 10th revision (ICD-10, 1995 and thereafter). Follow-up was conducted through December 2012. Incidents of cause-specific death were defined by ICD coding as follows: all cardiovascular disease, codes 393–459 (I00–I99) and non-cardiovascular disease, codes 001–392 and 460–999 (A00–H95 and J00–Y89).

### Measurement of parameters

Urinary protein, serum creatinine, and other factors were measured during the baseline survey when the participants were examined in 1993 [9]. Serum creatinine was measured automatically (RX-30, Nihon Denshi Inc., Tokyo, Japan) by using a modification of Jaffe’s reaction. GFR was estimated by using the modification of Diet in Renal Diseases (MDRD) method with the Japanese coefficient, as follows: eGFR (ml/min/1.73 m^2^) = 0.881 × 186.3 × age^-0.203^ × serum creatinine level^–1.154^ (× 0.742, if women) [10, 11]. Urinary protein was checked by dipstick urinalysis (Ames Hemacombisticks, Bayer–Sankyo, Tokyo, Japan), and trace-positive samples of proteinuria were re-examined by using the sulfosalicylic acid test; a result of ‘1+’ or more was regarded as proteinuria. Serum total cholesterol was measured by using an enzyme-based method. Other biochemical parameters used in risk analyses were measured as previously reported [9]. Blood pressure was measured by trained practitioners using standard mercury sphygmomanometry on the right arm of seated participants. Study participants were interviewed to ascertain the history and status regarding cigarette smoking, alcohol consumption, and stroke and heart diseases.

### Statistical analysis

Differences in age-adjusted mean values and the prevalence of potential confounding factors among patients in five eGFR categories (≥60, 50–59, 40–49, 30–39, and <30 ml/min/1.73 m^2^) were evaluated by analysis of covariance and chi-squared testing, respectively. The hazard ratio (HR) and its 95% confidence interval were calculated relative to the risk for people with eGFR ≥60 ml/min/1.73 m^2^. These estimates were adjusted for age and other potential confounding factors by using the Cox proportional hazards model. Potential confounding factors were age, presence of proteinuria, body mass index, hypertension category (normal blood pressure, high-normal blood pressure, grade 1 hypertension (mild), grade 2 hypertension or more (moderate and severe)) [12], anti-hypertensive treatment (yes or no), blood glucose level, diabetes treatment (yes or no), log-transformed serum triglyceride level, serum cholesterol level, and HDL cholesterol level, and use of lipid-lowering drugs (yes or no), cigarette smoking (never-smokers, ex-smokers, current 1–19 and ≥ 20 no./day), usual alcohol consumption (never, occasional, current <56 g/day, and ≥56 g/day in ethanol). All statistical analyses were conducted by using SAS, version 9.4 (SAS Institute, Cary, NC, USA)

## Results

**Table 1**presents mean ages and age-adjusted means and proportions of risk factors at baseline year, according to eGFR category. The percentage of men in the study population who comprised each eGFR subpopulation was 88.6% for ≥60 ml/min/1.73 m^2^, 9.5% for 50–59 ml/min/1.73 m^2^, 1.5% for 40–49 ml/min/1.73m^2^, 0.3% for 30–39 ml/min/1.73m^2^, and 0.1% for <30 ml/min/1.73 m^2^; for women, these percentages were 80.5%, 17.1%, 2.0%, 0.3%, and 0.1%, respectively.

**Table 1:** Age-adjusted mean values for patient characteristics at baseline year according to eGFR category.

		Estimated glomerular filtration rate (ml/min/1.73m^2^)					
		60≤	50–59	40–49	30–39	30>	*P* for trend
Men							
Number	(persons)	26904	2870	456	96	39	
Age	(years)	59.5	65.3	67.9	70.1	66.5	<0.001
Body mass index	(kg/m^2^)	23.2	23.8	24.0	23.8	22.6	<0.001
Systolic blood pressure	(mmHg)	135.9	139.7	141.5	143.2	144.2	<0.001
Diastolic blood pressure	(mmHg)	80.8	82.2	82.4	82.7	83.0	<0.001
Blood glucose	(mg/dl)	116.3	122.5	124.0	129.0	118.6	<0.001
Total cholesterol	(mg/dl)	192.5	196.4	196.3	192.4	184.5	<0.001
High-density lipoprotein	(mg/dl)	52.9	49.5	47.6	47.4	47.8	<0.001
Triglycerides	(mg/dl)	148.3	159.5	165.5	155.2	148.5	<0.001
Antihypertensive drug use	(%)	17.6	33.1	45.6	56.3	59.0	<0.001
Diabetic Treatment	(%)	3.4	5.4	7.9	10.4	12.8	<0.001
Lipid-lowering drug use	(%)	1.1	2.2	1.8	6.3	2.6	<0.001
Current smoker	(%)	52.4	42.6	38.2	40.7	56.4	<0.001
Daily alcohol consumption	(%)	54.0	39.3	32.9	37.5	28.2	<0.001
Women							
Number	(persons)	47639	10104	1165	204	70	
Age	(years)	55.8	65.7	66.4	69.1	65.8	<0.001
Body mass index	(kg/m^2^)	23.5	24.0	24.3	24.4	23.8	<0.001
Systolic blood pressure	(mmHg)	130.5	137.4	137.9	141.1	144.4	<0.001
Diastolic blood pressure	(mmHg)	77.4	79.1	79.3	80.0	79.8	<0.001
Blood glucose	(mg/dl)	106.4	115.3	118.7	126.4	117.5	<0.001
Total cholesterol	(mg/dl)	206.1	214.5	213.9	215.0	207.5	<0.001
High-density lipoprotein	(mg/dl)	57.3	55.0	53.5	51.2	48.5	<0.001
Triglycerides	(mg/dl)	130.4	151.3	155.8	171.4	167.5	<0.001
Antihypertensive drug use	(%)	17.6	33.1	45.6	56.3	59.0	<0.001
Diabetic Treatment	(%)	1.8	3.6	4.3	5.9	8.6	<0.001
Lipid-lowering drug use	(%)	2.7	5.1	5.3	3.9	8.6	<0.001
Current smoker	(%)	5.0	4.1	5.9	5.4	8.5	<0.001
Daily alcohol consumption	(%)	3.8	2.6	2.5	1.5	0.0	<0.001

*P* values were obtained by multiple regression analysis of eGFR levels adjusted for age.

Compared with people with eGFR ≥60 ml/min/1.73 m^2^ at baseline, the mean age of those with eGFR 30–39 ml/min/1.73 m^2^ was 11 years older for men and 13 years older for women. eGFR was inversely associated with systolic blood pressure, diastolic blood pressure, and body mass index (BMI). However, neither the mean age nor mean BMI of people in the lowest eGFR category was the highest among all eGFR categories. In addition, the eGFR category was inversely associated with the proportion of antihypertensive drug use, diabetes treatment, and current smokers but positively associated with alcohol consumption.

**Fig 1:**
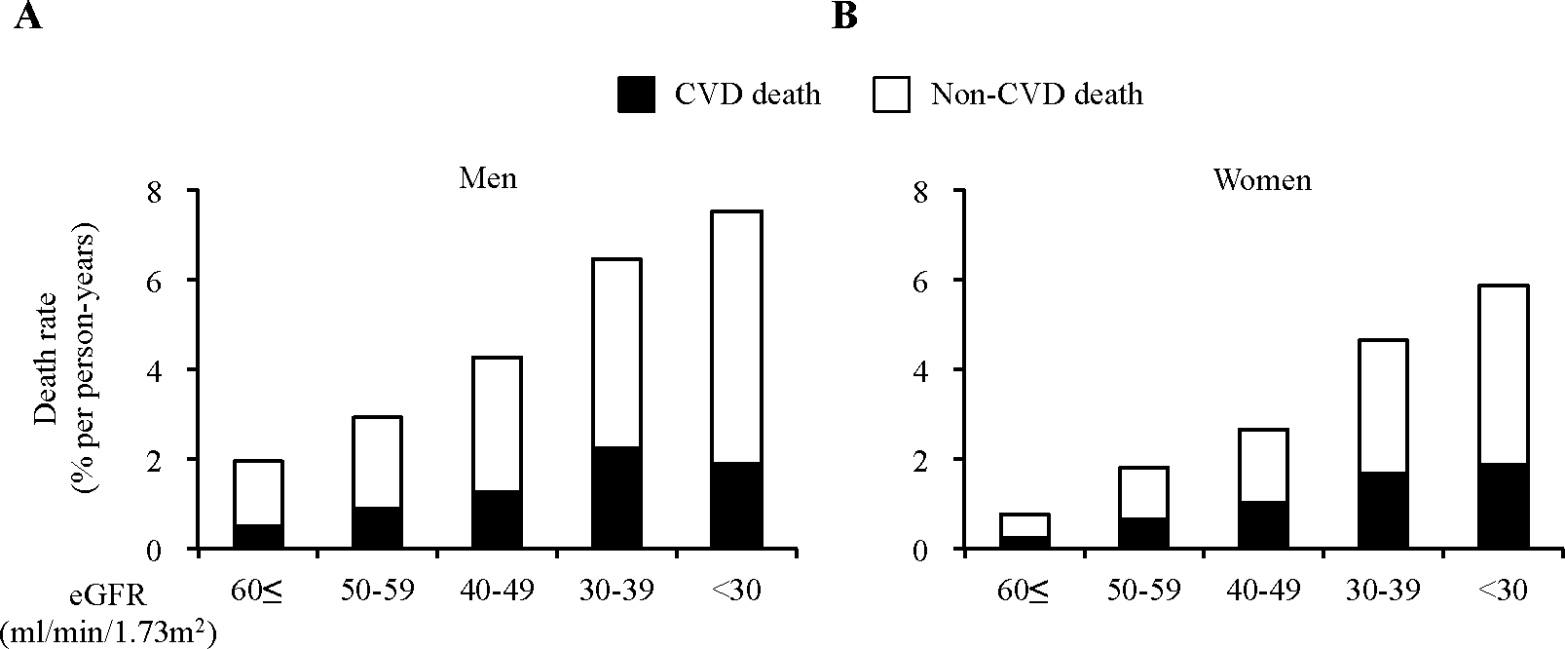
Distributions of cardiovascular disease (CVD) and non-CVD mortality rates according to estimated glomerular filtration rate (eGFR) in Japanese men and women. Distributions of CVD deaths and non-CVD deaths in men (A) and in women (B) were categorized according to eGFR in increments of 10 ml/min/1.73 m^2^.

The number of all-cause deaths was 20,534 (10,375 for men and 10,159 for women); that for CVD deaths was 5,995 (2,695 for men and 3,300 for women) and that for non-CVD deaths was 14,539 (7,680 for men and 6,859 for women). Dividing the study population by the eGFR revealed the increment in mortality according to the progression of CKD (**Fig 1A and 1B**). The numbers of CVD death in each eGFR category among follow-up years was 2,189, 395, 79, 25, 7 in men and 1,964, 1,088, 187, 46, 15 in women.

**Fig 2:**
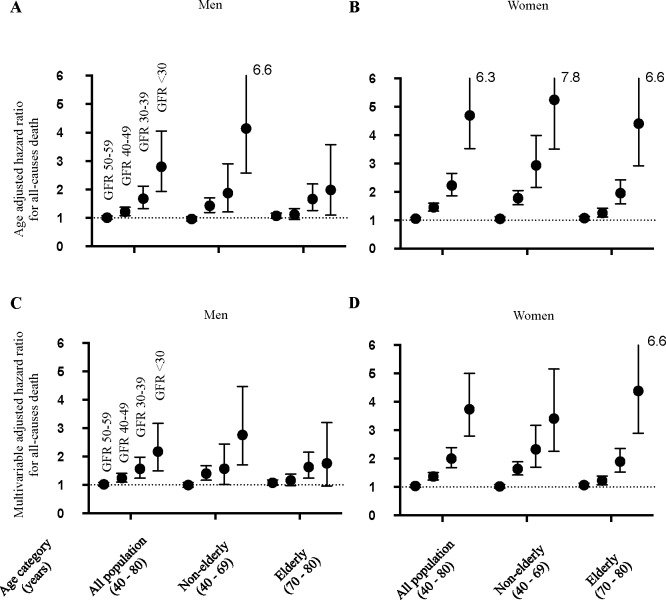
Risk of all-cause mortality according to estimated glomerular filtration rate (eGFR) and age category. Shown are age-adjusted (A and B) and multivariable-adjusted (C and D) hazard ratios with 95% confidence intervals for all-cause death with categorization according to eGFR in increments of 10 ml/min/1.73 m^2^ for subjects of all ages, non-elderly subjects, or elderly men (A and C) and women (B and D). Each hazard ratio was calculated relative to the subpopulation with eGFR ≥60 ml/min/1.73 m^2^. Adjusted factors for all-cause death were age, body mass index, urinary protein concentration, blood pressure, use of anti-hypertensive drugs, serum triglyceride concentration, serum high-density lipoprotein concentration, serum total cholesterol concentration, use of lipid-lowering drugs, blood glucose concentration, treatment for diabetes, smoking, and alcohol consumption.

**Fig 2**presents the HRs for all-cause death according to eGFR categorization as shown in **Table 1**. Compared with people with eGFR ≥60 ml/min/1.73 m^2^ as the reference group, most people with renal impairment were at significant risk for all-cause mortality after both age-adjustment (**Fig 2A and 2B**) and multivariable-adjustment, including for presence of proteinuria (**Fig 2C and 2D**). However, age- and multivariable-adjusted HRs were not significant in either men or women with eGFR 50–59 ml/min/1.73 m^2^. In contrast, the risk of all-cause death increased according to the progression of CKD among subjects with eGFR <50 ml/min/1.73 m^2^. When we further examined the HRs for all-causes mortality with categorization by age as non-elderly (40–69 years old) and elderly (70–80 years old), both age groups showed the same trend toward increased risk of all-causes mortality relative to the severity of CKD.

**Fig 3:**
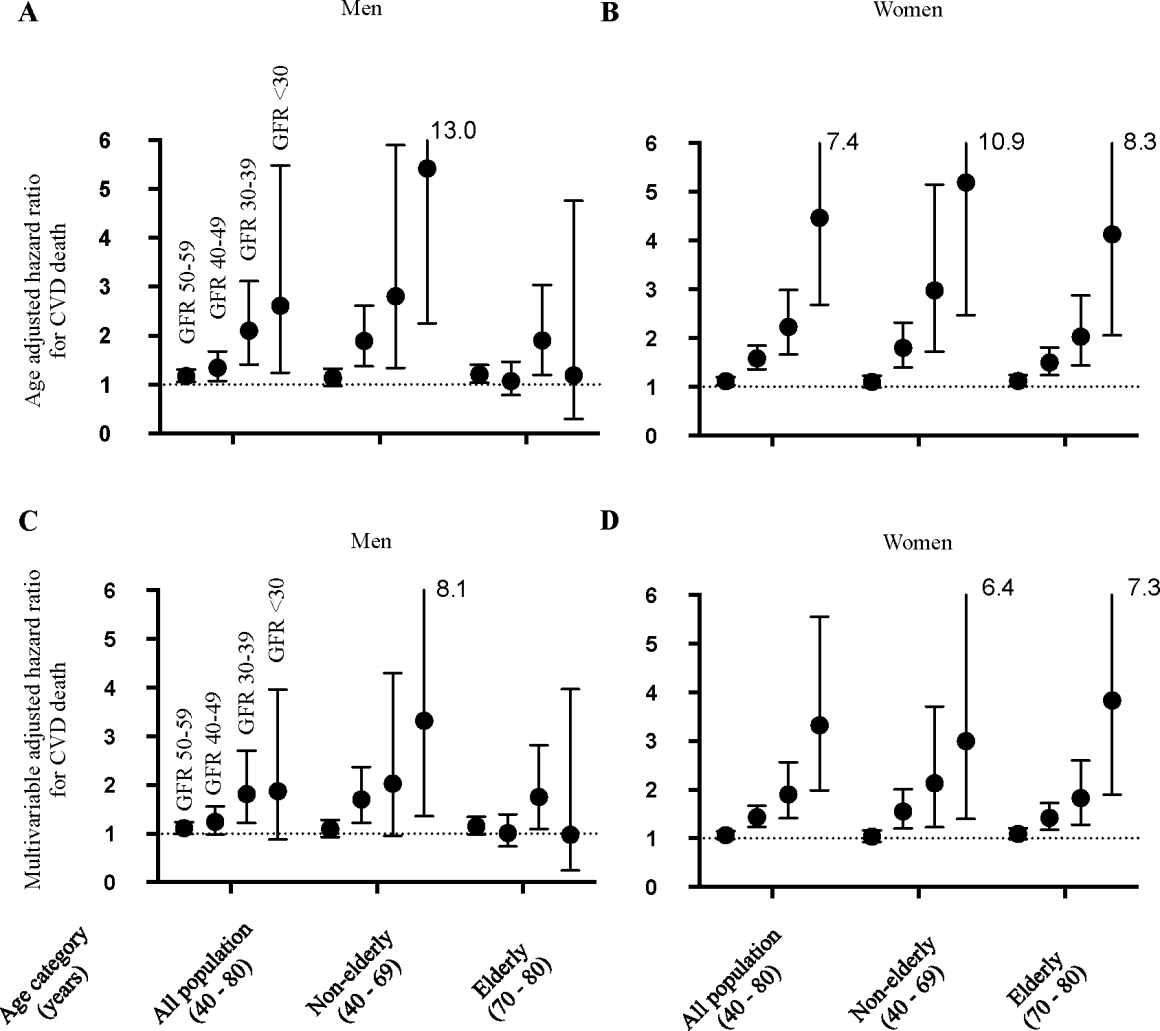
Risk of cardiovascular disease (CVD) mortality according to estimated glomerular filtration rate (eGFR) and age category. Shown are age-adjusted (A and B) and multivariable-adjusted (C and D) hazard ratios with 95% confidence intervals for CVD death with categorization according to eGFR in increments of 10 ml/min/1.73 m^2^ for subjects of all ages, non-elderly subjects, or elderly men (A and C) and women (B and D). Each hazard ratio was calculated relative to the subpopulation with eGFR ≥60 ml/min/1.73 m^2^. Adjusted factors for CVD death were age, body mass index, urinary protein concentration, blood pressure, use of anti-hypertensive drugs, serum triglyceride concentration, serum high-density lipoprotein concentration, serum total cholesterol concentration, use of lipid-lowering drugs, blood glucose concentration, treatment for diabetes, smoking, and alcohol consumption.

**Fig 3**presents the HRs for death due to CVD events according to the eGFR categorization shown in **Table 1**. Most of the age- and multivariable-adjusted HRs were not significant in men or women with eGFR 50–59 ml/min/1.73m^2^, and elderly men with eGFR 40–49 ml/min/1.73 m^2^ had no increased risk of CVD (*P >* 0.05). However, except for elderly men, subjects with eGFR <50 ml/min/1.73 m^2^ showed increased risk for CVD death according to the severity of CKD.

The current definition of CKD stage G3a is eGFR 45–59 ml/min/1.73 m^2^ and of G3b is 30–44 ml/min/1.73 m^2^, and subjects in the CKD 3a category are considered to be at lower risk of mortality than are those in CKD 3b. **Table 2**presents the HRs for death from all-causes or CVD events in subjects with eGFR 45–49 or 30–44 ml/min/1.73 m^2^ compared with those with eGFR ≥60 ml/min/1.73m^2^ as the reference. Multivariable-adjusted HR for CVD death in the eGFR 45–49 ml/min/1.73 m^2^ category was significantly increased (1.82; 95% confidential interval, 1.23–2.69) in non-elderly men, whereas CVD mortality in elderly men with eGFR 45–49 ml/min/1.73m^2^ were non significant. In contrast, both in non-elderly women and in elderly women with eGFR 45–49 ml/min/1.73 m^2^ showed significantly increases in the risks of all-cause mortality and CVD mortality.

**Table 2:** Risks of all-cause mortality and cardiovascular disease (CVD) mortality among subjects in CKD G3 category relative to those with eGFR ≥50 ml/min/1.73m^2^.

		Estimated glomerular filtration rate (ml/min/1.73 m^2^)					
		45–49			30–44		
Men		*n =* 312			*n =* 240		
	Adjustment	HR	95% CI	*P*	HR	95% CI	*P*
All causes							
All ages	Age	1.10	(0.94–1.28)		1.59	(1.36–1.85)	***
	Multivariable	1.10	(0.95–1.29)		1.58	(1.35–1.85)	***
40–69 y	Age	1.32	(1.05–1.66)	*	1.70	(1.34–2.17)	***
	Multivariable	1.33	(1.06–1.67)	*	1.53	(1.20–1.96)	***
70–80 y	Age	1.01	(0.82–1.24)		1.56	(1.27–1.90)	***
	Multivariable	1.02	(0.82–1.25)		1.63	(1.33–2.00)	***
CVD							
All ages	Age-	1.37	(1.05–1.78)	*	1.62	(1.21–2.17)	**
	Multivariable-	1.25	(0.96–1.63)		1.47	(1.09–1.98)	*
40–69 y	Age-	1.95	(1.32–2.88)	***	2.06	(1.31–3.24)	**
	Multivariable-	1.82	(1.23–2.69)	**	1.65	(1.04–2.62)	*
70–80 y	Age-	1.12	(0.78–1.59)		1.42	(0.96–2.08)	
	Multivariable-	1.03	(0.72–1.48)		1.37	(0.93–2.02)	
Women		*n =* 877			*n =* 492		
	Adjustment	HR	95% CI		HR	95% CI	
All causes							
All ages	Age	1.38	(1.23–1.54)	***	1.88	(1.66–2.12)	***
	Multivariable	1.31	(1.17–1.47)	*	1.71	(1.51–1.93)	***
40–69	Age	1.61	(1.37–1.90)	***	2.62	(2.14–3.20)	***
	Multivariable	1.50	(1.27–1.78)	***	2.21	(1.81–2.71)	***
70–80 y	Age	1.21	(1.04–1.41)	*	1.58	(1.36–1.84)	***
	Multivariable	1.19	(1.02–1.38)	*	1.53	(1.31–1.79)	***
CVD							
All ages	Age	1.51	(1.26–1.80)	***	1.94	(1.60–2.37)	***
	Multivariable	1.38	(1.15–1.66)	***	1.70	(1.39–2.07)	***
40–69 y	Age	1.51	(1.11–2.06)	**	2.92	(2.07–4.11)	***
	Multivariable	1.34	(0.98–1.82)		2.24	(1.58–3.17)	***
70–80 y	Age	1.50	(1.20–1.88)	***	1.69	(1.33–2.16)	***
	Multivariable	1.43	(1.14–1.79)	**	1.57	(1.23–2.00)	***

*, *P* < 0.05

**, *P* < 0.01

***, *P* < 0.001

CI, confidence interval

HR, hazard ratio.

## Discussion

Timely referral of CKD patients for specialized treatment is necessary to ensure informed decision-making and favorable outcomes both in terms of ESKD and of CVD [6]. However, precisely when patients should be referred for in-depth follow-up has not yet been determined and depends on a variety of factors [13]. The KDIGO guidelines note that people with CKD G3 and negativity for albuminuria need to be monitored but do not always need to be referred. In addition, the authors of a study evaluating a large cohort of Veterans Affairs patients cautioned that mortality risk assessment in elderly patients should not be based on the same eGFR cutoff points as those used for younger age groups and would benefit from finer categorization of the 30- to 59-ml/min/1.73 m^2^ eGFR group [5]. The KDIGO guidelines advocated the categorization of CKD according to cardiovascular outcomes [6, 7]. In this regard, CKD stage G3 was subdivided into G3a and G3b by applying a cutoff point of eGFR 45 ml/min/1.73 m^2^ for CVD risk assessment [7]. Therefore, we focused on patients with moderately decreased renal function as CKD G3 (eGFR 30–59 ml/min/1.73 m^2^) in the current study.

Our report published before the KDIGO 2012 guideline showed that not only CKD (<60 ml/min/1.73 m^2^) alone, but also CKD with proteinuria, is a risk factor for CVD death [9]. Except for Japan, many cohort studies were described to show the association of renal insufficiency and cardiovascular diseases (CVD) [5, 7, 14–16]. Among them, renal function categorized by CKD staging was conducted in recent studies to observe graded association between reduced eGFR and risk of CVD [7, 16], and other researchers utilized stratification of eGFR at interval of 10-ml/min/1.73 m^2^ instead of CKD staging [5]. Though the relationship between presence of CKD and incidence of CVD in a Japanese cohort were clearly shown in a previous report [9, 17], stratification by eGFR and by age of the cohort was not conducted. Therefore, little evidence is available on which to base the risk assessment of CVD according to eGFR level and different age category in the population with moderately decreased renal function in Japan. A CKD patient-based Gonryo cohort (N = 2,692) with stratification by CKD stage that had short observation period (12 months) had 69 CVD events and 24 patients with all-cause mortality [18]. It might not been examined in CVD mortality, as well as events, risk by sex- and age-dependency [18].

As shown in the profile of our current study population (**Table 1**), the association of eGFR level with age was undoubted. Although age is a risk factor for decreased eGFR [4, 19, 20], other aging-related factors, including increased blood pressure and increased proportion of anti-hypertensive drug use, strongly confounded each other. Therefore, we performed age-adjusted risk analyses, which revealed that, in the context of impaired renal function, people with lower eGFR are at greater risk of CVD, consistent with our previous findings [9]. Furthermore, we separately analyzed subjects aged 40–69 years (namely, non-elderly) and 70–80 years (elderly) to propose appropriate age categorizations for managing the risk of CVD comorbid with CKD, as promoted in the JSN guidelines [8].

Prognosis after CVD events is related to GFR, with a significant rise in mortality when eGFR falls below 45 ml/min/1.73 m^2^ [15, 16, 21]. As shown through global research efforts, for a person with an albumin to creatinine ratio of <10 mg/g, the odds ratio for CVD is 1.5 when the eGFR is 45–59 ml/min/1.73m^2^ and 2.2 when the eGFR 30–44 ml/min/1.73 m^2^, compared with the reference at eGFR 90–104 ml/min/1.73 m^2^ [22]. Therefore, CKD staging by using the G3a and G3b substages would be useful for managing populations at high risk of CVD, because the CKD risks of G3a patients are incomparable with that of G3b. However, it also could lead to disregard the risk of CVD in CKD G3a and we had little information about the risk of CVD mortality in Japanese patients with CKD G3a. Based on the stratification of eGFR at interval of 10-ml/min/1.73 m^2^ instead of CKD staging [5], we found the considerable risk of CVD in non-elderly men with an eGFR of 45–49 ml/min/1.73 m^2^ (i.e. CKD G3a) in this research (**Table 2**).

Mostly, proportionate increases in mortality with decreasing eGFR in a Japanese CKD population were shown (**Figs 2 and 3**). The CVD risk in elderly men did not show an eGFR-dependent increment in CKD G3–G5 as clearly, probably because the small size (less than a hundred in each category) and the number of CVD death in this population hampered the analysis. Overall, our results from the CVD risk analyses may support the JSN statement advocating both age- and eGFR-dependent management of nephrologist referral of patients with moderately decreased renal function (CKD G3).

In showing a relationship between CVD mortality and CKD, a particular strength of our study is its use of a large cohort from the general population with long-term follow-up; in comparison, the Hisayama Study involved 2634 community members with 12 years of follow-up [17], and The Framingham Heart Study included 6233 adult participants with 15 years of follow-up [14].

Our study had several limitations. First, eGFR was determined on the basis of the serum creatinine measured by using Jaffe’s reaction, which is less accurate as a measure of renal function than are actual GFR measurements by using inulin or ^125^I-iothalamate. In the MDRD study cohort of patients with GFR G3a–G4 (GFR 15–59 ml/min/1.73 m^2^), the cystatin C level was strongly associated with both all-cause and CVD mortality, particularly in elderly participants [23]. However, applying cystatin C testing to a general population is not feasible. Second, our current risk analyses adjusted for proteinuria as determined by a semi-quantitative dipstick method. Measurement of albuminuria is preferable in assessing the risk of CVD, given that dipstick quantification of proteinuria is relatively insensitive for detecting low levels of urinary protein [24, 25]. Third, some of the information (including use of drugs, diabetic treatment and smoking and alcohol consumption) was obtained via self-reported questionnaire. Forth, detailed information is lack in classes of using drug including anti-hypertensive drugs that may contribute to hemodynamic effects on the glomerulus and to inhibit the incidence of CVD.

In conclusion, here we demonstrated proportionate increases in mortality with decreasing eGFR in a Japanese CKD population. Specifically, we revealed both age- and eGFR-dependent changes in the risk of all-cause mortality and CVD mortality. Like patients in the CKD G3b subgroup, non-elderly men with an eGFR of 45–49 ml/min/1.73 m^2^ (i.e. CKD G3a) are at considerable risk of CVD mortality.

## Supporting Information

S1 Table Comparison of background characteristics between the cohort and excluded subjects. (DOC)
