# Supplementary material for: Relationship between Estimated Glomerular Filtration Rate and Cardiovascular Mortality in a Japanese Cohort with Long-Term Follow-Up
Source: PLoS One. 2016 Jun 6;11(6):e0156792. doi: 10.1371/journal.pone.0156792 (PMC4894635; doi:10.1371/journal.pone.0156792)
Supplement: S1 Table — (DOC) [file pone.0156792.s001.doc]

| Men | | Cohort subjects | Excluded | *P* for trend |
| --- | --- | --- | --- | --- |
| Number | (persons) | 30365 | 2766 |  |
| Age | (years) | 60.2 | 66.4 | <0.001 |
| Body mass index | (kg/m^2^) | 23.3 | 23.5 | 0.0108 |
| Systolic blood pressure | (mmHg) | 136.4 | 139.3 | <0.001 |
| Diastlic blood pressure | (mmHg) | 81.0 | 80.5 | 0.0303 |
| Blood glucose | (mg/dl) | 117.1 | 122.0 | <0.001 |
| Total cholesterol | (mg/dl) | 192.9 | 193.4 | 0.4707 |
| High-density lipoprotein | (mg/dl) | 52.5 | 51.6 | 0.0034 |
| Triglycerides | (mg/dl) | 149.7 | 144.4 | <0.001 |
| Antihypertensive drug use | (%) | 19.6 | 43.6 | <0.001 |
| Diabetic Treatment | (%) | 3.7 | 7.0 | <0.001 |
| Lipid-lowering drug use | (%) | 1.2 | 3.5 | <0.001 |
| Current smoker | (%) | 51.3 | 35.9 | <0.001 |
| Daily alcohol consumption | (%) | 52.2 | 41.0 | <0.001 |
| Women | | Cohort subjects | Excluded | *P* for trend |
| Number | (persons) | 59182 | 4730 |  |
| Age | (years) | 57.7 | 64.5 | <0.001 |
| Body mass index | (kg/m^2^) | 23.6 | 24.2 | <0.001 |
| Systolic blood pressure | (mmHg) | 131.8 | 136.5 | <0.001 |
| Diastlic blood pressure | (mmHg) | 77.8 | 78.6 | <0.001 |
| Blood glucose | (mg/dl) | 108.2 | 114.5 | <0.001 |
| Total cholesterol | (mg/dl) | 207.7 | 208.5 | 0.1775 |
| High-density lipoprotein | (mg/dl) | 56.8 | 55.6 | <0.001 |
| Triglycerides | (mg/dl) | 134.6 | 144.3 | <0.001 |
| Antihypertensive drug use | (%) | 19.4 | 42.7 | <0.001 |
| Diabetic Treatment | (%) | 2.2 | 4.0 | <0.001 |
| Lipid-lowering drug use | (%) | 3.2 | 6.3 | <0.001 |
| Current smoker | (%) | 4.9 | 4.3 | 0.2593 |
| Daily alcohol consumption | (%) | 3.5 | 2.4 | <0.001 |

**S1 Table 1**

**Comparison of background characteristics between the cohort and excluded subjects.**
